# Association of chronic pulmonary obstructive disease (COPD) and complications in head and neck surgery

**DOI:** 10.1590/S1808-86942011000100021

**Published:** 2015-10-19

**Authors:** Rogério Campos Cintra Volpe, Rafael Fitipaldi, Richard A Wessler P. Silva, Carlos Neutzling Lehn, Antonio Sérgio Fava

**Affiliations:** 1 Cidade de São Paulo University. Scholarship fund from the Scientific Initiation Institutional Progral (Programa Institucional de Iniciação Cientifica or PIIC); 2 Medical resident in the Head & Neck Surgery Unit, Public Servants State Hospital (Hospital do Servidor Público Estadual or HSPE-IAMSPE); 3 Otorhinolaryngologist, master's degree student, graduate program on Health Sciences, HSPE-IAMSPE; 4 Surgeon of the Head & Neck Surgery Unit, the Francisco Morato de Oliveira Public Servants State Hospital, São Paulo, SP; 5 Tutor in the PIIC. Head of the Head & Neck Surgery Unit, Public Servants State Hospital - IAMSPE

**Keywords:** postoperative complications, head and neck neoplasms, pulmonary disease, chronic obstructive

## Abstract

COPD presents in a variety of forms patients with head and neck cancer; it may affect therapeutic decision-making or postoperative outcomes due to its complications.

**Aim:** To correlate the severity of COPD in patients with head and neck SCC treated with surgery, who present postoperative complications.

**Method:** A retrospective analysis of 31 patients undergoing en bloc resections, from 2008 to 2009. All cases were evaluated and classified using the GOLD scale. The COPD grade, intubation period, ICU stay and hospital stay were studied.

**Results:** The mean age was 64.8 years; COPD was mild in 24 cases, moderate in 6 and severe in 1 case. ICU stay was 2.7 days and the intubation period was 1,12 days. The mean hospital stay was 24.4 days. There was no relation between COPD grade and brochopneumonia, intubation period, ICU stay and hospital stay.

**Conclusion:** Patients with head and neck SCC have a tendency to acquire COPD; its severity was not related with postoperative pulmonary complications, prolonged intubation period, ICU stay and hospital stay.

## INTRODUCTION

Tobacco consumption is closely related with head and neck cancer and chronic obstructive pulmonary disease (COPD).^1,^^2^ The frequency of COPD (grades I to IV) has increased 4 to 9% worldwide.^3^ Smoking is recognized at present as a risk factor for several chronic diseases. COPD and head and neck cancer are strongly associated with smoking; this relation increases with duration of exposure and the lifelong tobacco load of individuals.^4^, ^5^, ^6^

Head and neck cancer, and specifically squamous cell carcinoma of the upper aerodigestive tract, is one of the most common cancers in the world population. Its treatment requires a multidisciplinary approach comprising surgery, radiotherapy and chemotherapy, especially at more advanced stages of disease.^2,^^7^ Head and neck cancer is treated with surgery, radiotherapy, chemotherapy, or any combination of these.^8^ These approaches usually cause significant morbidity to patients; other concomitant diseases hinder necessary therapy, at times even contraindicating the required treatment. This reality may affect the clinical progression or the survival of these patients.^9,^^10^

The incidence of head and neck cancer is highest in the elderly population. These patients may require major surgery as one of the treatment options; if lung function is compromised, they are at a higher risk of developing severe pulmonary complications, which may entail longer hospital or intensive care unit (ICU) stays, more time intubated, use of wide spectrum antibiotics, and other measures. The preoperative evaluation is therefore extremely important; studies have shown that when well carried out, the intraoperative period is safer, and there are fewer postoperative complications. Postoperative pulmonary complications are common and are one of the main causes of morbidity and mortality. Clinically significant complications include atelectasis, infection (bronchitis and e pneumonia), respiratory failure, exacerbated chronic lung disease, and bronchospasm. Risk factors for these complications are emergency surgery, age over 50 years, surgery lasting over three house, prolonged mechanical ventilation, poor health status as defined by ASA class over 2, congestive heart failure, COPD, PaCO2>45 mmHg, an abnormal chest X-ray, cigarette smoking within the past 8 weeks, upper airway infection, and the need for a nasogastric tube postoperatively.^1,^^11,^^12^

We believe that COPD is present to some degree in head and neck squamous cell carcinoma cases because the same risk factors are present in both categories of patients. Thus, the presence of COPD may alter clinical decision-making since it entails specific complications.

## OBJETIVE

The purpose of this study was to correlate COPD grade with the incidence of postoperative complications in patients with head and neck squamous cell carcinoma following surgery.

## MATERIALS AND METHODS

A retrospective study was carried out in the form of a review of registries of patients submitted to en bloc surgery (removal of the primary tumor and neck dissection) for the treatment of head and neck squamous cell carcinoma from 01 February 2008 to 31 January 2009. All patients had been evaluate preoperatively at the Respiratory Disease Unit in the same hospital and classified according to COPD grade based on Gold's scale (Table 1).

**Table 1:** Spirometry criteria for COPD severity according to Gold

I: mild COPD	FEV1/CVF < 0.7	In this stage patients may not be aware of abnormal lung function.
	FEV1 ≥ 80% of predicted	
II: moderate COPD	FEV1/CVF < 0.7	Symptoms develop, such as breathlessness, typically with effort.
	50% ≤ FEV1 < 80% of predicted	
III: severe COPD	FEV1/CVF < 0.7	Breathlessness typically worsens and often limits daily activities. Exacerbation begins to appear.
	30% ≤ FEV1 < 50% of predicted	
IV: very severe COPD	FEV1/CVF < 0.7	The quality of life is significantly affected and exacerbation may place life at risk.
	FEV1 < 30% of predicted OR FEV1 < 50% of predicted associated with acute respiratory failure	

Antibiotic prophylaxis (ceftriaxone and clindamycin) was given to all patients at the time of induction of anesthesia and maintained until 48 hours after surgery. These drugs were continued at therapeutic doses in patients that developed postoperative infection. Patients were sent to the ICU for the immediate postoperative period. Correlations between COPD and duration of orotracheal intubation, ICU stay, and postoperative hospital stay were evaluated. Patients with incomplete files were excluded from the study. The χ^2^ test or Fisher's exact test were applied for quantitative variables. The Kaplan-Meier and the log-rank tests were applied to analyze the time elapsed until removal of tubes, ICU stay, and postoperative hospital stay. The MedCalc (release 11.1.1, Mariakerke, Belgium) software was used for the statistical analysis.

## RESULTS

This review comprised 31 medical files. The mean age of patients was 64.8 years (33-83 years). The maleto-female ratio was 8:1. The mean tobacco load was 34.7 (0-100) packs per year. All patients had some grade of COPD. Table 2 presents the incidence of COPD according to Gold's classification.

**Table 2:** Incidence of COPD according to Gold's classification

Classification	Number of patients	%
Grade I (mild)	24	77,4
Grade II (moderate)	06	19,4
Grade III (severe)	01	03,2
Grade IV (very severe)	00	00,0
Total	31	100

The mean immediate postoperative ICU stay was 2.7 days (1-12 days). The mean duration of orotracheal intubation was 1.12 days (0-9 days). The mean hospital stay was 24.4 days (4-70 days).

Bronchopneumonia developed postoperatively in 4 patients (16.1%). There were no other respiratory complications.

**Figure 1:**
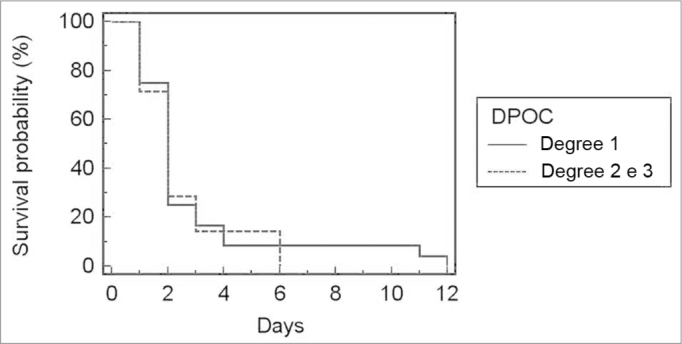
Relationship between the degree of COPD and ICU stay.

**Figure 2:**
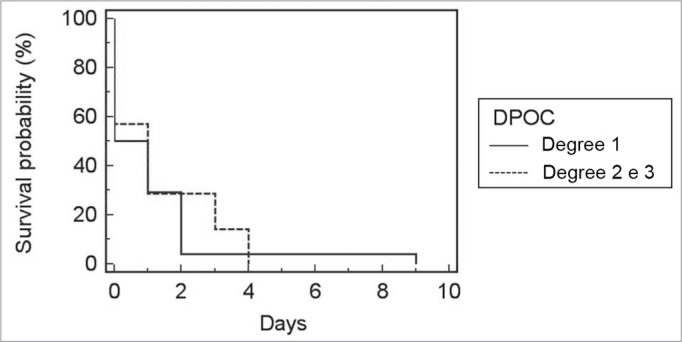
Relationship between the degree of COPD and orotracheal intubation.

**Figure 3:**
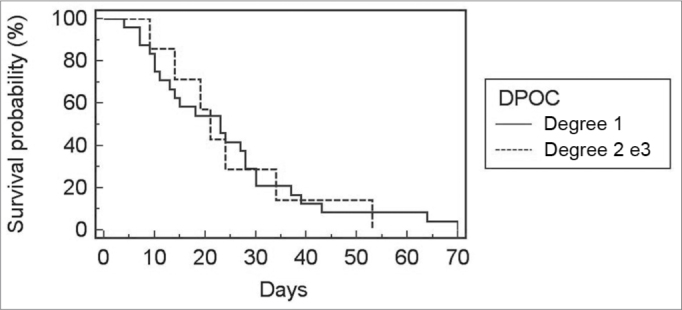
Relationship between COPD and hospital stay.

Because only one patient had COPD grade 3, this group was included in the group of COPD grade 2 patients. There was no correlation between COPD grade and the incidence of bronchopneumonia (p=0.66), duration of orotracheal intubation (Fig. 1; *p*=0.71), ICU stay (Fig. 2; *p*=0.79), and postoperative hospital stay (Fig. 3; *p*=0.91).

## DISCUSSION

COPD and head and neck cancer - particularly upper aerodigestive tract tumors - are frequently associated, since smoking is a risk factor for both disease. COPD may place constraints on the indication of specific procedures for treating head and neck cancers; for instance, the indication of partial laryngectomies may be limited.^13^, ^14^, ^15^ Aspiration is always present in these procedures regardless of how much of the larynx has been resected. The “sphincter” function of the larynx is compromised to some degree even in more limited partial laryngectomies. This is of particular importance in COPD patients, in which ventilatory of respiratory capacity is limited to some degree. Depending on the degree of lung involvement, surgery may be contraindicated because of the risk of aspiration.^16^, ^17^, ^18^, ^19^

Our series has the usual features of head and neck cancer patients: the mean age was 64.8 years, and males predominated.^7,^^19^ Similarly, most of the patients had mild grade COPD, of which patients are often unaware because of the paucity of symptoms.^2^

What stood out in the results was the presence of bronchopneumonia in 16.1% of cases. This number raises questions even in a small sample (31 cases in the present study). Pulmonary atelectasis is often seen in patients undergoing general anesthesia with mechanical ventilation. It is more frequent in surgeries of the thoracic and/or abdominal cavities for clear reasons (restricted postoperative ventilation because of pain and hypoventilated lung segments, longer time in the hospital bed, etc.). These factors may be excluded head and neck en bloc surgery cases, since most patients are able to walk on the next day after surgery, and the thorax and abdomen are not surgically manipulated.

Tracheostomy is frequent in patients operated for the treatment of upper aerodigestive tract tumors. This could be considered a risk factor for lower respiratory infection; but arguments could be raised because of decreased dead space and risk of aspiration, as well as easier bronchopulmonary hygiene.

As there was no significant difference between COPD grade and duration of orotracheal intubation and hospital stay, we may focus on a common situation for all patients: immediate postoperative ICU stay and mechanical ventilation beyond the duration of anesthesia. The rate of bronchopneumonia may have been different if patients had been sent to semi-intensive care without mechanical ventilation (only tracheostomy as needed depending on the surgery). Further, the number of case in the present study is small; in a future study, as a larger sample becomes possible, we may be able to stratify by procedure, and thereby demonstrate a better association between COPD and complications of surgery.

## CONCLUSION

Patients with squamous cell carcinoma of the upper aerodigestive tract are at a high risk for developing COPD of any grade. The severity of COPD was unrelated to the incidence of postoperative pulmonary complications or to the need for prolonged intubation, ICU stay or hospital stay.
